# Evolutionary Relationships of Microbial Aromatic Prenyltransferases

**DOI:** 10.1371/journal.pone.0027336

**Published:** 2011-11-30

**Authors:** Tobias Bonitz, Vikram Alva, Orwah Saleh, Andrei N. Lupas, Lutz Heide

**Affiliations:** 1 Pharmaceutical Institute, Eberhard Karls-Universität Tübingen, Tübingen, Germany; 2 Department of Protein Evolution, Max-Planck-Institute for Developmental Biology, Tübingen, Germany; University of South Florida College of Medicine, United States of America

## Abstract

The linkage of isoprenoid and aromatic moieties, catalyzed by aromatic prenyltransferases (PTases), leads to an impressive diversity of primary and secondary metabolites, including important pharmaceuticals and toxins. A few years ago, a hydroxynaphthalene PTase, NphB, featuring a novel ten-stranded β-barrel fold was identified in *Streptomyces* sp. strain CL190. This fold, termed the PT-barrel, is formed of five tandem ααββ structural repeats and remained exclusive to the NphB family until its recent discovery in the DMATS family of indole PTases. Members of these two families exist only in fungi and bacteria, and all of them appear to catalyze the prenylation of aromatic substrates involved in secondary metabolism. Sequence comparisons using PSI-BLAST do not yield matches between these two families, suggesting that they may have converged upon the same fold independently. However, we now provide evidence for a common ancestry for the NphB and DMATS families of PTases. We also identify sequence repeats that coincide with the structural repeats in proteins belonging to these two families. Therefore we propose that the PT-barrel arose by amplification of an ancestral ααββ module. In view of their homology and their similarities in structure and function, we propose to group the NphB and DMATS families together into a single superfamily, the PT-barrel superfamily.

## Introduction

Aromatic prenyltransferases (PTases) catalyze the transfer of isoprenyl moieties to aromatic acceptor molecules, forming C-C bonds. They are key enzymes in the biosynthesis of lipoquinones and of many secondary metabolites in plants, fungi and bacteria [1].

**Figure 1:**
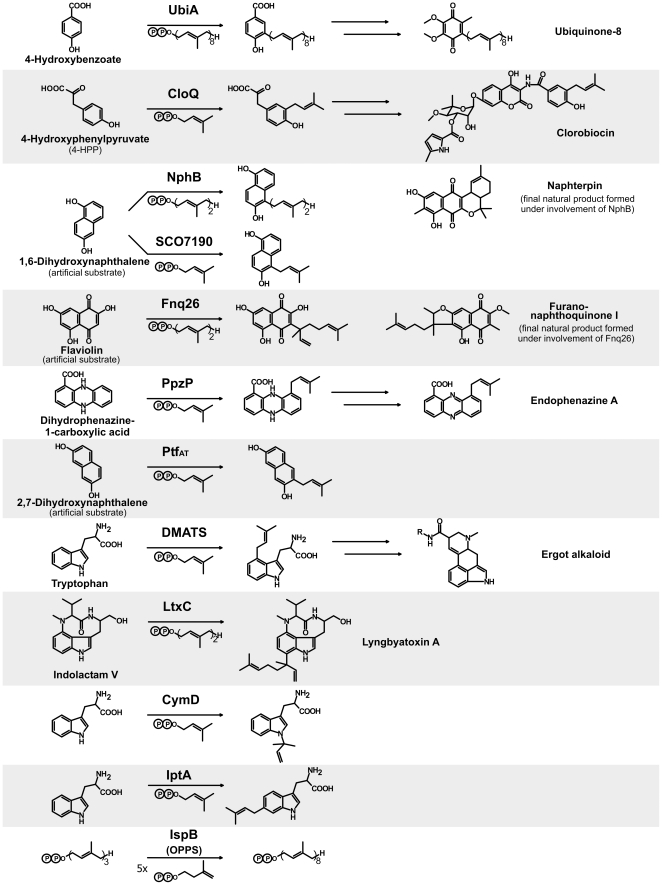
Reactions catalyzed by aromatic prenyltransferases and by the octaprenyl diphosphate synthase IspB.

Aromatic PTases of lipoquinone biosynthesis are integral membrane proteins. They contain an aspartate-rich motif (e.g. NDxxD) for binding of the prenyl diphosphate substrate *via* a Mg^2+^ ion, similar to the corresponding motif of farnesyl diphosphate synthase [2]. A structural model of the PTase UbiA involved in the biosynthesis of ubiquinone (Fig. 1) has been proposed [3].

**Figure 2:**
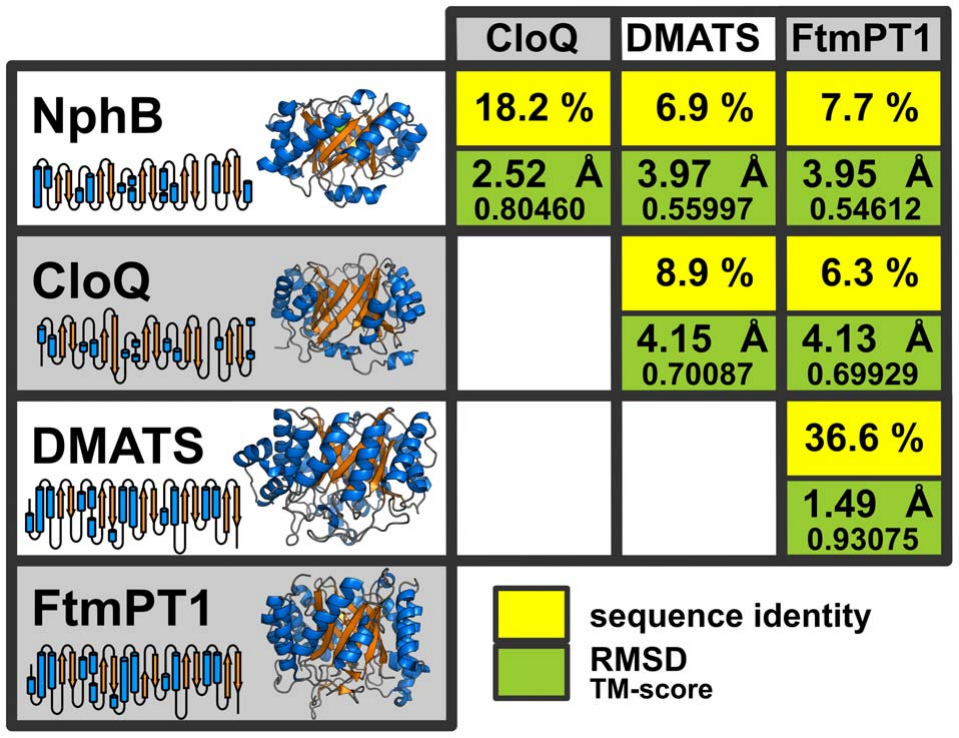
Structures of PT-barrels. Pairwise sequence identities, RMSDs and TM-scores of the four aromatic prenyltransferases NphB (PDB 1ZB6), CloQ (2XLQ), DMATS (3I4Z) and FtmPT1 (3O2K) are shown. The schemes besides the structures depict the topology of the secondary structural elements.

In contrast to the PTases of lipoquinone biosynthesis, the aromatic PTase CloQ from *Streptomyces roseochromogenes*, involved in the formation of clorobiocin (Fig. 1), was found to be a soluble, monomeric 35 kDa protein [4]. CloQ does not contain a NDxxD motif and is active in the absence of Mg^2+^ or other divalent cations. Kuzuyama et al. [5] identified a similar aromatic prenyltransferase, NphB, involved in the biosynthesis of the prenylated polyketide naphterpin (Fig. 1) in *Streptomyces* sp. strain CL190. NphB was found to display a hitherto unobserved β-barrel fold which was termed the PT-barrel (Fig. 2; PDB 1ZB6). It consists of five repetitive ααββ elements. The ten β-strands arrange in an antiparallel fashion to form a central β-barrel that contains the active center in its spacious lumen and the α-helices form a solvent-exposed ring around the barrel [6]. The structure of the aforementioned CloQ also displays the PT-barrel fold (Fig. 2; PDB 2XLQ) [7]. PSI-BLAST searches currently reveal 17 further database entries with sequence similarity to NphB and CloQ, 12 of them in bacteria of the genus *Streptomyces* and five in fungi of the phylum *Ascomycota*. In silico structure predictions suggest that all these proteins adopt the PT-barrel fold. Eleven of these enzymes have been investigated biochemically, and all of them catalyze the *C*-prenylation of aromatic compounds, i.e. phenols or phenazines. Fig. 1 shows as examples the reactions catalyzed by the bacterial enzymes CloQ, NphB, SCO7190, Fnq26 and PpzP, and by the fungal enzyme Ptf_At_. Tab. S1 (Supplementary Material) lists the references and accession numbers for all these enzymes and for all other proteins included in this study.

The *C*-prenylation of an aromatic compound is also catalyzed by dimethylallyltryptophan synthase (DMATS; Fig. 1), involved in the biosynthesis of the pharmaceutically important ergot alkaloids in different fungi of the phylum *Ascomycota*. DMATS shows no sequence similarity, as evaluated with PSI-BLAST, to the bacterial enzyme NphB or orthologs thereof, and is considerably larger than NphB (459 vs. 307 amino acids). Unexpectedly, however, it was found to adopt the same PT-barrel fold as NphB (Fig. 2; PDB 3I4X) [8]. DMATS is the prototype of the fungal indole PTases, involved in the biosynthesis of a large number of complex secondary metabolites in fungi [9]. Currently, approximately 200 close orthologs of DMATS are found in different fungal genomes in the database. The structure of a second member of this group, FtmPT1, has recently been published, and it shows the same fold as DMATS (Fig. 2; PDB 3O2K) [10]. Furthermore, three indole PTases (LtxC, CymD and IptA; Fig. 1) have recently been identified in bacteria [11], [12], [13], and GenBank currently contains 16 further entries from bacterial genomes with similarity to these three enzymes. Most of these entries are found in genomes of actinomycetales, but one is from the alphaproteobacterium *Methylobacterium* sp. 4–46, and LtxC is from a cyanobacterium. In silico structure prediction suggests that all these bacterial indole PTases adopt the PT-barrel fold.

A similarity in sequence between the phenol/phenazine PTases (NphB/CloQ family) and the indole PTases (DMATS/CymD family) is not detectable using BLAST and PSI-BLAST. This raises the question whether the NphB/CloQ family and the DMATS/CymD family may have originated independently and converged on the PT-barrel fold in response to the biochemical challenge of performing an aromatic prenylation reaction, i.e. a reaction corresponding to a Friedel-Crafts alkylation, in an aqueous solution which requires effective shielding of the reactive intermediary allylic cation from reaction with water [8].

Only a limited number of structural solutions is available to a polypeptide chain, therefore protein structures are multiply convergent [14]. In contrast, sequence space is essentially infinite and many sequences are compatible with a particular fold. For this reason, sequence similarity rather than structure similarity is the primary marker of homology. In the recent years, the enormous growth of protein sequence and structure databases coupled with the development of sensitive sequence comparison methods has shown that proteins may not be as polyphyletic as hitherto assumed [15]. Indeed, many fold families, for instance families of the TIM (βα)_8_-barrel fold, that were previously considered to be analogous are now thought to be homologous [16], [17], [18].

In this study, we used a highly sensitive sequence comparison method, HHsearch [19], based on profile hidden Markov Models (HMMs) to evaluate the evolutionary origins of the CloQ/NphB and the DMATS/CymD families. Our results indicate that they are homologous. We also include an investigation on the membrane-bound aromatic PTases, e.g. of lipoquinone biosynthesis. They show no evolutionary relationship to the CloQ/NphB and the DMATS/CymD families but display evolutionary connections to other PTases such as protoheme IX farnesyltransferases, chlorophyll a synthases and decaprenyl-phosphate 5-phosphoribosyltransferases.

## Materials and Methods

To calculate the root mean square deviation (RMSD) and TM-scores for the proteins included in this study, we used the TM-align server (http://zhanglab.ccmb.med.umich.edu/TM-align/) [20] with default parameters. To evaluate homology between PTases we used HHsearch [21], a sensitive remote homology detection method based on the pairwise comparison of profile hidden Markov models (HMMs). HHsearch was used to perform all-against-all comparison of the 36 biochemically investigated proteins listed in supplementary Table S1. For each of these 36 proteins, we generated multiple sequence alignments using the buildali script from the HHsearch package. The obtained multiple alignments were used to calculate profile HMMs using HHmake, also from the HHsearch package. The profile HMMs were compared with each other using HHsearch and the results were mapped onto a matrix. All tools were run using default settings.

To gather the amino acid sequences of PTases for cluster analysis, we searched the non-redundant protein sequence database (nr) at NCBI for homologs of NphB from *Streptomyces* sp. strain CL190 (PDB identifier 1ZB6) and DMATS from *Aspergillus fumigatus* (3I4X) using the PSI-BLAST algorithm [22] in four iterative steps. The sequences which were shorter than 200 amino acid residues and longer than 600 amino acid residues were excluded to avoid fragments and multi-domain proteins. The same procedure was applied to search for homologs of UbiA from *Escherichia coli* (AP_004541), MenA from *E. coli* (AAB01207) and Slr1736 from *Synechocystis* sp. PCC 6803 (BAA17774) in four consecutive PSI-BLAST iterations. All identified sequences were pooled together and duplicates were removed using RetrieveSeq tool from the MPI bioinformatic toolkit (http://toolkit.tuebingen.mpg.de) [23]. The sequence XP_003295160 was removed due to the presence of unidentified amino acids in the sequence.

All identified sequences were analyzed and clustered by their pairwise PSI-BLAST P-values [24] with CLANS (http://toolkit.tuebingen.mpg.de/clans; [25]). CLANS treats sequences as point masses in a virtual space which attract or repel each other depending on their pairwise sequence similarities. Clustering was done to equilibrium in 2D at a P-value cutoff of 1E-3 for the cluster map of NphB and DMATS, and 1E-6 for the UbiA, MenA and Slr736 cluster map using default settings.

To detect sequence repeats in PT-barrels, we used the highly sensitive de novo repeat detection method HHrepID [26]. HHrepID takes a multiple sequence alignment as input and converts it into a profile HMM. To detect internal sequence repeats, this profile HMM is repeatedly aligned to itself. We extracted sequences of bacterial and fungal indole PTases, and phenol/phenazine PTases from the aforementioned cluster map and calculated multiple sequence alignments with ClustalW [27]. These alignments were then analyzed for the presence of repeats with HHrepID using default settings.

## Results

*HMM-HMM comparisons of PTases featuring the PT-barrel:* Sequence search methods achieve different levels of sensitivity, depending on the amount of information they incorporate. Sequence-to-sequence methods, such as BLAST [24], are the least sensitive as they use only the information from the pairwise comparison of two sequences, scored by a global substitution matrix. Profile-to-sequence methods, such as the iterated version of BLAST, PSI-BLAST [28], are more sensitive, as they include family-specific information for the query sequence in the form of a position-specific scoring matrix derived from homologous sequences. Profile-to-profile comparison methods, such as COMPASS [29], provide an additional improvement by using family-specific information for both sequences being compared. Incorporation of position-specific gapping probabilities into the profiles yields profile Hidden Markov Models (HMMs) [30], which are currently our most sensitive tool for the detection of sequence similarity. HHsearch [19], [21], an HMM-to-HMM comparison method, has a sensitivity comparable to that of advanced fold recognition methods, despite using only sequence information.

**Figure 3:**
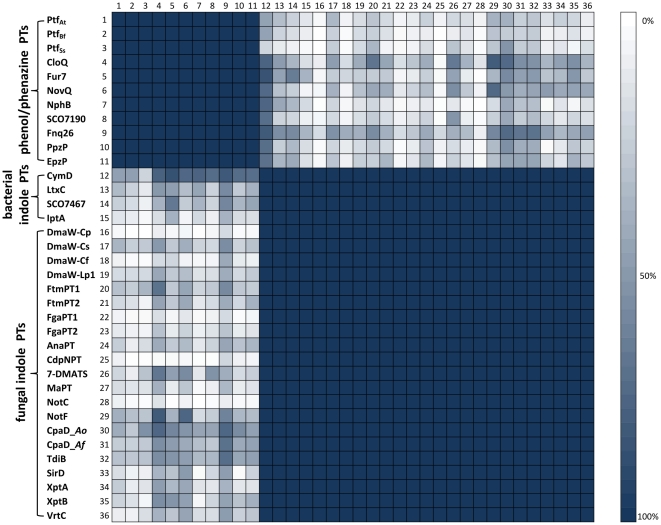
HHsearch analysis of prenyltransferases with the PT-barrel fold. Pairwise HMM comparisons of 36 biochemically characterized PTases (see Table S1, Supplementary Material) were performed using HHsearch. Group and protein names are shown on the left. Cell color indicates HHsearch probability of the match as depicted in the scale on the right.

While members of the CloQ/NphB and the DMATS/CymD families display the PT-barrel fold (the structures of NphB and DMATS align at a RMSD of 3.97 Å over 290 aligned residues), they show very little sequence identity (<15%). Nevertheless many instances are known where proteins with such low sequence identity belong to the same superfamily (e.g. ubiquitins [31]). We therefore used HHsearch to investigate the evolutionary origins of these two families. Biochemically characterized members of (i) the PTases with similarity to NphB/CloQ, (ii) the fungal indole PTases and (iii) the bacterial indole PTases were selected as representatives for HHsearch analysis. As expected, HHsearch assigns a 100% probability of homology to all pairwise matches within each of these three groups (Fig. 3). We also detected matches between the fungal indole PTases (e.g. DMATS) and the bacterial indole PTases (e.g. CymD) at a probability of 100%, confirming their evolutionary relatedness. Likewise, we also obtained probability values of 100% for connections between the bacterial phenol PTases NphB and CloQ, and the fungal phenol PTases Ptf_At_, Ptf_Bf_ and Ptf_Sc_. Strikingly, we obtained several matches between the CloQ/NphB and the DMATS/CymD families at high probabilities (50%–75%). We have previously shown that this level of sequence similarity is indicative of common ancestry [32], [33], [34], [35]. We thus conclude that these two families are homologous.

In the biosynthesis of ubiquinones, menaquinones, plastoquinones and tocopherols, the *C*-prenylation of aromatic substrates is catalyzed by integral membrane proteins with several membrane-spanning helices [1]. Similar to the soluble farnesyl diphosphate synthase (FPP synthase) [2] and the octaprenyl diphosphate synthase IspB [36] (Fig. 1), these membrane-bound aromatic PTases show conserved NDxxD motifs for the binding of the isoprenoid substrates in the form of Mg^2+^ complexes. In contrast, all aromatic PTases characterized by the PT-barrel fold are soluble enzymes without the NDxxD motifs. As expected, HHsearch detected matches between the membrane-bound aromatic PTases UbiA of ubiquinone biosynthesis, MenA of menaquinone biosynthesis and Str1736 of tocopherol biosynthesis, confirming their homology (data not shown). In contrast, these enzymes did not make any connections to the soluble PTases with the PT-barrel fold.

To check for the existence of possible distant homologs of the aromatic PTases with the PT-barrel fold, we ran HHsearch against a database comprising several complete genomes. The search was seeded with the PTases NphB and DMATS. We did not find matches to proteins outside of the CloQ/NphB and the DMATS/CymD families, indicating that the PT-barrel fold is exclusive to them at this time.

*Detection of sequence repeats in the PT-barrel:* The PT-barrel is a toroidal fold, in which five ααββ structural repeats are arranged in a circular fashion to form a closed barrel. While these five repeats are structurally well superimposable with median RMSDs below 2.5 Å, they do not show clear sequence similarity to each other. Therefore, it has remained unclear whether the symmetry displayed by the PT-barrel is a result of five-fold amplification of a single ααββ unit or of structural convergence. If PT-barrels originated by amplification, we might still find residual sequence similarity between their repeats with highly sensitive sequence comparison tools. For this, we used the de novo repeat detection method HHrepID, which detects internal sequence symmetries by repeatedly aligning the query HMM with itself. HHrepID has been used successfully to detect highly divergent sequence repeats in several folds including TIM (βα)_8_-barrels [18] and outer membrane β-barrels [34]. We detected five-fold internal sequence symmetry in both the bacterial and the fungal indole PTases at default settings with a P-value of better than 1E-4. We also found repeats in the phenol/phenazine PTases, albeit at lower detection stringency. In the indole PTases the detected repeats coincide largely with the ααββ structural units, but in the phenol/phenazine PTases the repeats are shorter and coincide only with the ββ hairpins. While we can substantiate a scenario for the origin of indole PTases by amplification based on the presence of residual sequence similarity between their repeats, the repeats of phenol/phenazine PTases are more divergent and a scenario for their origin cannot be established at this time. We note that this range of internal symmetry among members of the same superfamily is not unique to PT-barrels. β-propellers, for instance, display a wide range of internal symmetry, from near-identical to fully diverged, and an origin by amplification has been proposed for them [35].

**Figure 4:**
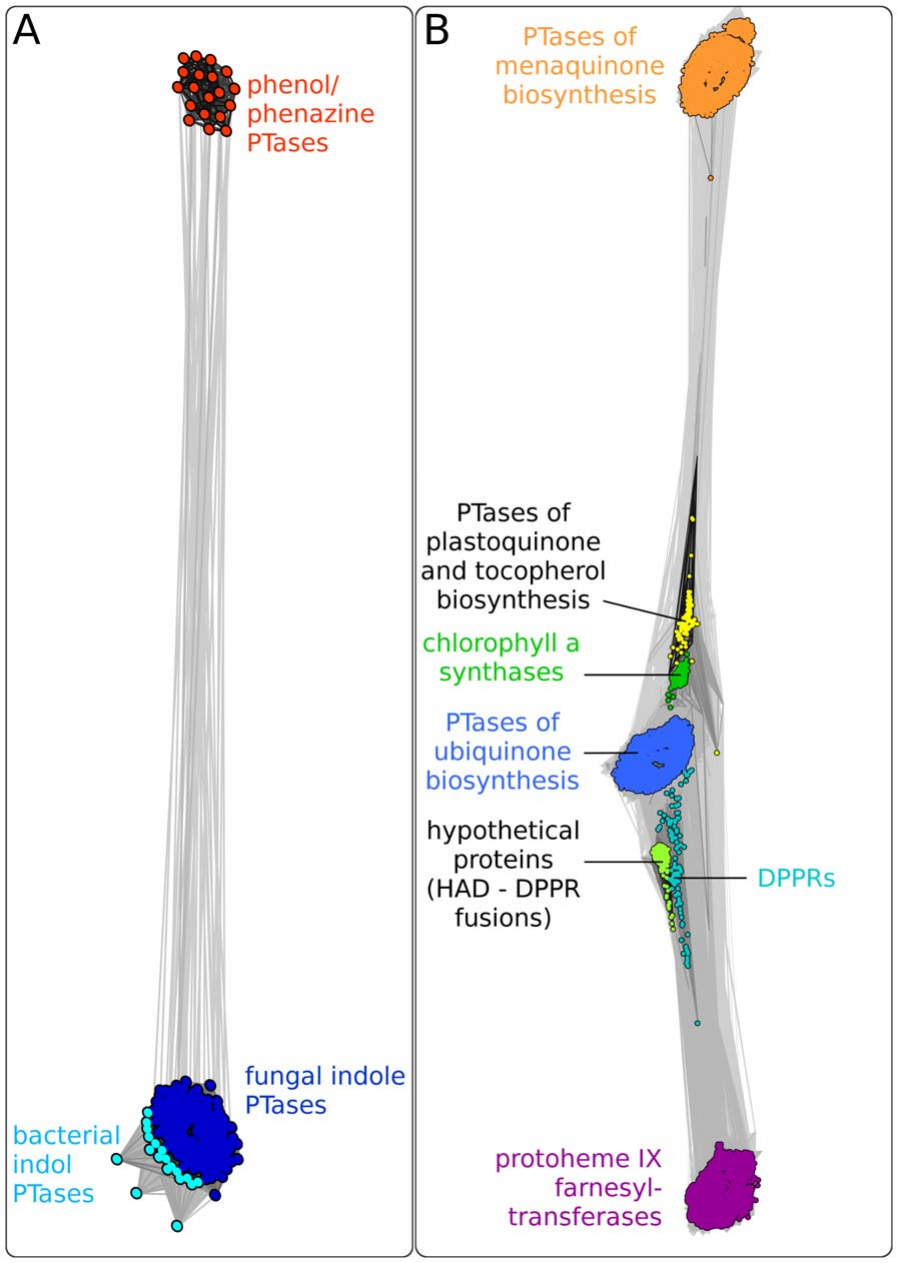
Cluster map of (A) aromatic prenyltransferases characterized by the PT-barrel fold and (B) membrane-bound aromatic prenyltransferases. These maps were generated by clustering respective PTases in CLANS based on their all-against-all pairwise similarities as measured by PSI-BLAST P-values. Dots represent sequences, line coloring reflects PSI-BLAST P-values; the darker a line, the lower the P-value.

*Cluster analysis of aromatic PTases:* In order to visualize the relationships between the PTases with the PT-barrel fold, we searched the non-redundant protein sequence database at NCBI for homologs of NphB and DMATS and clustered the obtained sequences in CLANS. The resulting cluster map (Fig. 4A) very clearly shows two distinct clusters that correspond to the phenol/phenazine PTases and the indole PTases. The two clusters are connected with each other, further confirming the proposed evolutionary relationship between these two enzyme families. No other groups of proteins with similarity to NphB and DMATS were identified by this PSI-BLAST search, showing that the enzymes with PT-barrel fold are not related to other currently known proteins.

The phenol/phenazine PTases (Fig 4A; dark orange) comprise 14 bacterial proteins from the genus *Streptomyces* and 5 fungal proteins from the phylum *Ascomycota.* The cluster analysis did not show a separation of the bacterial and the fungal enzymes within this family, even at higher clustering stringency. In contrast, the indole PTases can be separated into two subclusters, one of which contains all of the 19 bacterial entries, and the other one all of the 186 fungal entries. This separation is already visible in Fig. 4A, and becomes very clear at higher clustering stringency (data not shown).

We also performed a cluster analysis of the membrane-bound aromatic PTases. We searched the non-redundant protein sequence database at NCBI for homologs of the membrane-bound PTases UbiA, MenA and Slr1736 and clustered them in CLANS. As expected, the map (Fig. 4 B) shows distinct but connected clusters for (i) 4-hydroxybenzoate PTases of ubiquinone biosynthesis, e.g. UbiA of *E. coli* [37], (ii) 1,4-dihydroxy-2-naphthoate 3-prenyltransferases of menaquinone biosynthesis, e.g. MenA of *E. coli* [38], and (iii) homogentisate PTases of plastoquinone and tocopherol biosynthesis [39]. In addition, this cluster analysis revealed further enzymes to be related to the aromatic PTase of lipoquinone biosynthesis. These include the chlorophyll a synthases and protoheme IX farnesyltransferases, both of which attach phytyl or farnesyl moieties to side chains of tetrapyrrole substrates [40], [41]. Another group is formed by the 5-phosphoribose-1-diphosphate:decaprenyl-phosphate 5-phosphoribosyltransferases (DPPRs) which are involved in the biosynthesis of lipids of the bacterial cell wall. The reaction catalyzed by DPPRs is quite different from that catalyzed by aromatic PTases, yet there is obvious sequence similarity between DPPR and UbiA [42]. A last group of database entries related to membrane-bound aromatic PTases comprises hypothetical proteins, mostly from proteobacteria, which consist of two distinct domains: one similar to hydrolases of the HAD superfamily [43], the other one similar to DPPR or UbiA. The function of these proteins is, to our knowledge, unknown.

## Discussion

**Figure 5:**
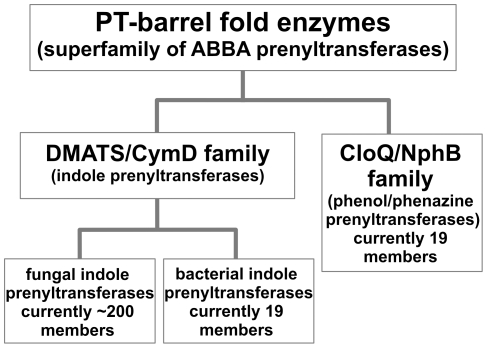
Classification of prenyltransferases characterized by the PT-barrel fold ( = “ABBA prenyltransferases”).

The PT-barrel is a novel protein fold that was discovered recently and is found exclusively in microbial secondary metabolic PTases with aromatic substrates. For proteins with the PT-barrel fold, the name ABBA PTases has been suggested previously [6], owing to the αββα succession of the secondary structure elements in the polypeptide chain which results in the characteristic antiparallel orientation of the β-sheets in the barrel. Our study suggests that all proteins with the PT-barrel fold share a common ancestry and they therefore belong to a single superfamily. As shown in Fig. 5, this superfamily can be divided into two families, i.e. the indole PTases and the phenol/phenazine PTases. The state-of-the-art sequence comparison method HHsearch yielded significant matches between these families, indicating a common ancestry. We also found evidence for the origin of the PT-barrel fold by amplification of an ancestral ααββ module.

The family of indole PTases comprises the fungal indole PTases and the bacterial indole PTases, with DMATS and CymD as typical representatives, respectively. It should be noted that the term “indole PTases” is correct for most but not all biochemically investigated members of this family. The exceptions are SirD (NCBI accession AAS92554), which catalyses the *O*-prenylation of the phenolic oxygen of tyrosine in sirodesmin biosynthesis [44], VrtC (ADI24928), which *C*-prenylates a phenolic substrate which is related to tetracyclines [45], and TdiB (ABU51603) which catalyses both an indole prenylation and the prenylation of a phenolic moiety during terrequinone biosynthesis A [46].

As expected, HHsearch did not indicate a relationship between the soluble aromatic PTases with the PT-barrel fold, such as NphB or DMATS, and the membrane-bound PTases, such as UbiA of ubiquinone biosynthesis. Therefore, two independent solutions have evolved in nature to solve the biochemical problem of catalyzing an aromatic prenylation reaction in an aqueous environment. The indispensable shielding of the reactive allylic cation, generated from the prenyl diphosphate substrate, is achieved by a barrel of antiparallel β-sheets in case of the ABBA PTases, and by a deep lipophilic pocket between the transmembrane helices in case of the membrane-bound aromatic PTases.

All PTases characterized by the PT-barrel fold belong to secondary metabolic pathways; no primary metabolic enzyme with this fold has been discovered yet. In contrast, most of the membrane-bound aromatic PTases are involved in primary metabolism. However, a few enzymes of this group are involved in secondary metabolism. The bacterial PTase AqgD catalyzes the *O*-prenylation of the secondary metabolite alkyl-methoxyhydroquinone [47], and the fungal PTase XP_751272 is involved in the biosynthesis of pyripyropene A [48]; both show similarity to UbiA of ubiquinone biosynthesis. The bacterial putative PTase BAD07390 is likely to be involved in the biosynthesis of the secondary metabolite BE-40644 [49]; it shows similarity to MenA of menaquinone biosynthesis. The recently characterized bacterial PTase AuaA is involved in the biosynthesis of Auracin D [50] and is located inbetween the UbiA and protoheme IX farnesyltransferase clusters in the map depicted in Fig. 4B.

During our cluster analysis of membrane-bound aromatic PTases, we noticed that many bacterial and fungal genomes contain not one but several genes for (biochemically not yet characterized) proteins annotated as “UbiA prenyltransferase” or similar. For instance, the genome of *Salinispora tropica* contains two genes annotated as “4-hydroxybenzoate polyprenyltransferase” (YP_001160901) and “UbiA PTase” (YP_001161073). The genome of *Catenulispora acidiphila* likewise contains a gene annotated as “4-hydroxybenzoate polyprenyltransferase” (YP_003118736) and in addition three “UbiA prenyltransferase” genes (YP_003112865, YP_003115669 and YP_003116365). Both organisms are Gram-positive bacteria which are believed to not produce ubiquinones [51]. It remains to be shown whether such UbiA-like enzymes may be involved in the biosynthesis of secondary metabolites. In plants several PTases with homology to enzymes of ubiquinone and plastoquinone biosynthesis have recently been shown to be involved in the biosynthesis of important secondary metabolites [52].

Both the membrane-bound and the soluble aromatic PTases show remarkable promiscuity for their aromatic substrates and have been used for the chemoenzymatic synthesis of new prenylated aromatic compounds [53], [54], [55], [56], [57], [58]. Protein engineering has allowed altering the substrate specificity of indole PTases [9], [59]. Therefore, these PTases may represent promising tools for biotechnological and pharmaceutical research.

## Supporting Information

Table S1 Proteins included in this study.
(PDF)

## References

[1] Heide L (2009). Prenyl transfer to aromatic substrates: genetics and enzymology.. Curr Opin Chem Biol.

[2] Poulter C (2006). Farnesyl diphosphate synthase. A paradigm for understanding structure and function relationships in *E*-polyprenyl diphosphate synthases.. Phytochem Rev.

[3] Bräuer L, Brandt W, Schulze D, Zakharova S, Wessjohann L (2008). A structural model of the membrane-bound aromatic prenyltransferase UbiA from *E. coli*.. Chembiochem.

[4] Pojer F, Wemakor E, Kammerer B, Chen H, Walsh CT (2003). CloQ, a prenyltransferase involved in clorobiocin biosynthesis.. Proc Natl Acad Sci U S A.

[5] Kuzuyama T, Noel JP, Richard SB (2005). Structural basis for the promiscuous biosynthetic prenylation of aromatic natural products.. Nature.

[6] Tello M, Kuzuyama T, Heide L, Noel JP, Richard SB (2008). The ABBA family of aromatic prenyltransferases: broadening natural product diversity.. Cell Mol Life Sci.

[7] Metzger U, Keller S, Stevenson CE, Heide L, Lawson DM (2010). Structure and mechanism of the magnesium-independent aromatic prenyltransferase CloQ from the clorobiocin biosynthetic pathway.. J Mol Biol.

[8] Metzger U, Schall C, Zocher G, Unsöld I, Stec E (2009). The structure of dimethylallyl tryptophan synthase reveals a common architecture of aromatic prenyltransferases in fungi and bacteria.. Proc Natl Acad Sci U S A.

[9] Steffan N, Grundmann A, Yin WB, Kremer A, Li SM (2009). Indole prenyltransferases from fungi: a new enzyme group with high potential for the production of prenylated indole derivatives.. Curr Med Chem.

[10] Jost M, Zocher G, Tarcz S, Matuschek M, Xie X (2010). Structure-function analysis of an enzymatic prenyl transfer reaction identifies a reaction chamber with modifiable specificity.. J Am Chem Soc.

[11] Edwards DJ, Gerwick WH (2004). Lyngbyatoxin biosynthesis: sequence of biosynthetic gene cluster and identification of a novel aromatic prenyltransferase.. J Am Chem Soc.

[12] Schultz AW, Lewis CA, Luzung MR, Baran PS, Moore BS (2010). Functional characterization of the cyclomarin/cyclomarazine prenyltransferase CymD directs the biosynthesis of unnatural cyclic peptides.. J Nat Prod.

[13] Takahashi S, Takagi H, Toyoda A, Uramoto M, Nogawa T (2010). Biochemical characterization of a novel indole prenyltransferase from *Streptomyces* sp. SN-593.. J Bacteriol.

[14] Cheng H, Kim B-H, Grishin NV (2008). MALISAM: a database of structurally analogous motifs in proteins.. Nucleic Acids Res.

[15] Alva V, Remmert M, Biegert A, Lupas AN, Soding J (2010). A galaxy of folds.. Protein Sci.

[16] Copley RR, Bork P (2000). Homology among (betaalpha)(8) barrels: implications for the evolution of metabolic pathways.. J Mol Biol.

[17] Nagano N, Orengo CA, Thornton JM (2002). One fold with many functions: the evolutionary relationships between TIM barrel families based on their sequences, structures and functions.. J Mol Biol.

[18] Söding J, Remmert M, Biegert A (2006). HHrep: de novo protein repeat detection and the origin of TIM barrels.. Nucleic Acids Res.

[19] Söding J (2005). Protein homology detection by HMM-HMM comparison.. Bioinformatics.

[20] Zhang Y, Skolnick J (2005). TM-align: a protein structure alignment algorithm based on the TM-score.. Nucleic Acids Res.

[21] Söding J, Biegert A, Lupas AN (2005). The HHpred interactive server for protein homology detection and structure prediction.. Nucleic Acids Res.

[22] Altschul SF, Wootton JC, Gertz EM, Agarwala R, Morgulis A (2005). Protein database searches using compositionally adjusted substitution matrices.. FEBS Journal.

[23] Biegert A, Mayer C, Remmert M, Söding J, Lupas AN (2006). The MPI Bioinformatics Toolkit for protein sequence analysis.. Nucleic Acids Res.

[24] Altschul SF, Gish W, Miller W, Myers EW, Lipman DJ (1990). Basic local alignment search tool.. J Mol Biol.

[25] Frickey T, Lupas A (2004). CLANS: a Java application for visualizing protein families based on pairwise similarity.. Bioinformatics.

[26] Biegert A, Söding J (2008). De novo identification of highly diverged protein repeats by probabilistic consistency.. Bioinformatics.

[27] Larkin MA, Blackshields G, Brown NP, Chenna R, McGettigan PA (2007). Clustal W and Clustal X version 2.0.. Bioinformatics.

[28] Altschul SF, Madden TL, Schaffer AA, Zhang J, Zhang Z (1997). Gapped BLAST and PSI-BLAST: a new generation of protein database search programs.. Nucleic Acids Res.

[29] Sadreyev R, Grishin N (2003). COMPASS: a tool for comparison of multiple protein alignments with assessment of statistical significance.. J Mol Biol.

[30] Eddy SR (1998). Profile hidden Markov models.. Bioinformatics.

[31] Bayer P, Arndt A, Metzger S, Mahajan R, Melchior F (1998). Structure determination of the small ubiquitin-related modifier SUMO-1.. J Mol Biol.

[32] Alva V, Ammelburg M, Söding J, Lupas AN (2007). On the origin of the histone fold.. BMC Struct Biol.

[33] Kopec KO, Alva V, Lupas AN (2010). Homology of SMP domains to the TULIP superfamily of lipid-binding proteins provides a structural basis for lipid exchange between ER and mitochondria.. Bioinformatics.

[34] Remmert M, Biegert A, Linke D, Lupas AN, Söding J (2010). Evolution of outer membrane beta-barrels from an ancestral beta beta hairpin.. Mol Biol Evol.

[35] Chaudhuri I, Söding J, Lupas AN (2008). Evolution of the beta-propeller fold.. Proteins.

[36] Asai K, Fujisaki S, Nishimura Y, Nishino T, Okada K (1994). The identification of *Escherichia coli ispB* (cel) gene encoding the octaprenyl diphosphate synthase.. Biochem Biophys Res Commun.

[37] Melzer M, Heide L (1994). Characterization of polyprenyldiphosphate: 4-hydroxybenzoate polyprenyltransferase from *Escherichia coli*.. Biochim Biophys Acta.

[38] Suvarna K, Stevenson D, Meganathan R, Hudspeth ME (1998). Menaquinone (vitamin K2) biosynthesis: localization and characterization of the *menA* gene from *Escherichia coli*.. J Bacteriol.

[39] Savidge B, Weiss JD, Wong YH, Lassner MW, Mitsky TA (2002). Isolation and characterization of homogentisate phytyltransferase genes from *Synechocystis* sp. PCC 6803 and *Arabidopsis*.. Plant Physiol.

[40] Saiki K, Mogi T, Anraku Y (1992). Heme O biosynthesis in *Escherichia coli*: The *cyoE* gene in the cytochrome *bo* operon encodes a protoheme IX farnesyltransferase.. Biochem Biophys Res Commun.

[41] Oster U, Bauer CE, Rudiger W (1997). Characterization of chlorophyll a and bacteriochlorophyll a synthases by heterologous expression in *Escherichia coli*.. J Biol Chem.

[42] Huang H, Scherman MS, D'Haeze W, Vereecke D, Holsters M (2005). Identification and active expression of the *Mycobacterium tuberculosis* gene encoding 5-phospho-a-D-ribose-1-diphosphate: decaprenyl-phosphate 5-phosphoribosyltransferase, the first enzyme committed to decaprenylphosphoryl-D-arabinose synthesis.. J Biol Chem.

[43] Koonin EV, Tatusov RL (1994). Computer analysis of bacterial haloacid dehalogenases defines a large superfamily of hydrolases with diverse specificity. Application of an iterative approach to database search.. J Mol Biol.

[44] Zou HX, Xie X, Zheng XD, Li SM (2010). The tyrosine *O*-prenyltransferase SirD catalyzes *O*-, *N*-, and *C*-prenylations.. Appl Microbiol Biotechnol.

[45] Chooi YH, Cacho R, Tang Y (2010). Identification of the viridicatumtoxin and griseofulvin gene clusters from *Penicillium aethiopicum*.. Chem Biol.

[46] Balibar CJ, Howard-Jones AR, Walsh CT (2007). Terrequinone A biosynthesis through L-tryptophan oxidation, dimerization and bisprenylation.. Nat Chem Biol.

[47] Awakawa T, Fujita N, Hayakawa M, Ohnishi Y, Horinouchi S (2011). Characterization of the biosynthesis gene cluster for alkyl-*O*-dihydrogeranyl-methoxyhydroquinones in *Actinoplanes missouriensis*.. Chembiochem.

[48] Itoh T, Tokunaga K, Matsuda Y, Fujii I, Abe I (2010). Reconstitution of a fungal meroterpenoid biosynthesis reveals the involvement of a novel family of terpene cyclases.. Nat Chem.

[49] Dairi T (2005). Studies on biosynthetic genes and enzymes of isoprenoids produced by actinomycetes.. J Antibiot (Tokyo).

[50] Stec E, Pistorius D, Muller R, Li SM (2011). AuaA, a membrane-bound farnesyltransferase from Stigmatella aurantiaca, catalyzes the prenylation of 2-methyl-4-hydroxyquinoline in the biosynthesis of aurachins.. Chembiochem.

[51] Nowicka B, Kruk J (2010). Occurrence, biosynthesis and function of isoprenoid quinones.. Biochim Biophys Acta.

[52] Yazaki K, Sasaki K, Tsurumaru Y (2009). Prenylation of aromatic compounds, a key diversification of plant secondary metabolites.. Phytochemistry.

[53] Koeduka T, Shitan N, Kumano T, Sasaki K, Sugiyama A (2011). Production of prenylated flavonoids in tomato fruits expressing a prenyltransferase gene from *Streptomyces coelicolor* A3(2).. Plant Biol.

[54] Shindo K, Tachibana A, Tanaka A, Toba S, Yuki E (2011). Production of novel antioxidative prenyl naphthalen-ols by combinational bioconversion with dioxygenase PhnA1A2A3A4 and prenyltransferase NphB or SCO7190.. Biosci Biotechnol Biochem.

[55] Kumano T, Richard SB, Noel JP, Nishiyama M, Kuzuyama T (2008). Chemoenzymatic syntheses of prenylated aromatic small molecules using *Streptomyces* prenyltransferases with relaxed substrate specificities.. Bioorg Med Chem.

[56] Macone A, Lendaro E, Comandini A, Rovardi I, Matarese RM (2009). Chromane derivatives of small aromatic molecules: Chemoenzymatic synthesis and growth inhibitory activity on human tumor cell line LoVo WT.. Bioorg Med Chem.

[57] Ozaki T, Mishima S, Nishiyama M, Kuzuyama T (2009). NovQ is a prenyltransferase capable of catalyzing the addition of a dimethylallyl group to both phenylpropanoids and flavonoids.. J Antibiot (Tokyo).

[58] Xiao Y, Machacek M, Lee K, Kuzuyama T, Liu P (2009). Prenyltransferase substrate binding pocket flexibility and its application in isoprenoid profiling.. Mol Biosyst.

[59] Li SM (2009). Applications of dimethylallyltryptophan synthases and other indole prenyltransferases for structural modification of natural products.. Appl Microbiol Biotechnol.

