# Supplementary material for: Evolutionary Relationships of Microbial Aromatic Prenyltransferases
Source: PLoS One. 2011 Nov 30;6(11):e27336. doi: 10.1371/journal.pone.0027336 (PMC3227686; doi:10.1371/journal.pone.0027336)
Supplement: Table S1 — Proteins included in this study. (PDF) [file pone.0027336.s001.pdf]

SUPPLEMENTAL DATA FOR

Evolutionary Relationships of Microbial Aromatic Prenyltransferases

T. Bonitz, V. Alva, O. Saleh, A. N. Lupas, L. Heide

Table S1: Proteins included in this study

Name	organism	NCBI Accession	Reference for biochemical data
**NphB/CloQ family (phenol/phenazine prenyltransferases)**			
CloQ*	*Streptomyces roseochromogenes subsp. oscitans*	AAN65239	[1]
Fnq26*	*Streptomyces cinnamonensis*	CAL34104	[2]
Fur7*	*Streptomyces* sp. KO-3988	BAE78975	[3]
NphB*	*Streptomyces* sp. Strain Cl190	1ZB6_A	[4]
NovQ*	*Streptomyces niveus* DSM 40088	AAF67510	[5]
EpzP*	*Streptomyces cinnamonensis*	ADQ43372	[6]
PpzP*	*Streptomyces anulatus*	CAX48655	[7]
Ptf_{At}*	*Aspergillus terreus* NIH2624	EAU39467	[8]
Ptf_{Bf}*	*Botryotinia fuckeliana* B05.10	EDN25735	
Ptf_{Ss}*	*Sclerotinia sclerotiorum* 1980	EDN93598	
SCO7190*	*Streptomyces coelicolor* A3(2)	NP_631248	[9]
**DMATS/CymD family<br>a) fungal indole prenyltransferases**			
DmaW-Cp*	*Claviceps purpurea*	Q6X2E0	[10]
DmaW-Cs*	*Clavicipitaceae* sp. US2005a	AAZ29613	[11]
DmaW-Cf*	*Claviceps fusiformis*	AAC18893	[12]
DmaW-Lp1*	*Epichloe typhina* x *Neotyphodium lolii*	AAP81206	
FgaPT1*	*Aspergillus fumigatus* Af293	XP_756136	[13]
FgaPT2*	*Aspergillus fumigatus*	AAX08549/ 3I4Z_A	[14]
FtmPT1*	*Aspergillus fumigatus*	AAX56314/ 3O2K_A	[15]
FtmPT2*	*Aspergillus fumigatus*	ACF22981	[16]
CdpNPT*	*Aspergillus fumigatus*	ABR14712	[17]
AnaPT*	*Neosartorya fischeri* NRRL 181	EAW16181	[18]
7-DMATS*	*Aspergillus fumigatus*	ABS89001	[19]
TdiB*	*Emericella nidulans*	ABU51603	[20]
MaPT*	*Malbranchea aurantiaca* RRC1813	ABZ80612	[21]
NotC*	*Aspergillus* sp. MF297-2	ADM34131	[22]
NotF*	*Aspergillus* sp. MF297-2	ADM34132	
CpaD_*Ao**	*Aspergillus oryzae* RIB40	XP_001821505	[23]
CpaD_*Af**	*Aspergillus flavus* NRRL3357	XP_002379958	

XptA*	*Aspergillus nidulans* FGSC A4	XP_664388	[24]
XptB*		see reference	
SirD*	*Leptosphaeria maculans*	AAS92554	[25]
VrtC*	*Penicillium aethiopicum*	ADI24928	[26]
**DMATS/CymD family b) bacterial indole prenyltransferases**			
IptA* = 6-dimethylallyl-tryptophan synthase	*Streptomyces* sp. SN-593	BAJ07990	[27]
CymD* = Sare_4565	*Salinispora arenicola* CNS-205	YP_001539324	[28]
LtxC*	*Lyngbya majuscula*	AAT12285	[29]
SCO7467*	*Streptomyces coelicolor* A3(2)	NP_631515	--
**Prenyltransferases of lipoquinone biosynthesis**			
UbiA	*Escherichia coli* str. K-12 substr. W3110	AP_004541	[30]
MenA	*Escherichia coli*	AAB01207	[31]
Slr1736	*Synechocystis* sp. PCC 6803	BAA17774	[32]
UbiA	*Salinispora tropica* CNB-440	YP_001160901	--
UbiA	*Salinispora tropica* CNB-440	YP_001161073	--
UbiA	*Catenulispora acidiphila* DSM 44928	YP_003118736	--
UbiA	*Catenulispora acidiphila* DSM 44928	YP_003112865	--
UbiA	*Catenulispora acidiphila* DSM 44928	YP_003115669	--
UbiA	*Catenulispora acidiphila* DSM 44928	YP_003116365	--

* Used in HHpred analysis (Figure 3)

REFERENCES

1. Pojer F, Wemakor E, Kammerer B, Chen H, Walsh CT, et al. (2003) CloQ, a prenyltransferase involved in clorobiocin biosynthesis. Proc Natl Acad Sci U S A 100: 2316-2321.
2. Haagen Y, Glück K, Fay K, Kammerer B, Gust B, et al. (2006) A gene cluster for prenylated naphthoquinone and prenylated phenazine biosynthesis in *Streptomyces cinnamonensis* DSM 1042. Chembiochem 7: 2016-2027.
3. Kumano T, Tomita T, Nishiyama M, Kuzuyama T (2010) Functional characterization of the promiscuous prenyltransferase responsible for furaquinocin biosynthesis: identification of a physiological polyketide substrate and its prenylated reaction products. J Biol Chem.
4. Kuzuyama T, Noel JP, Richard SB (2005) Structural basis for the promiscuous biosynthetic prenylation of aromatic natural products. Nature 435: 983-987.
5. Ozaki T, Mishima S, Nishiyama M, Kuzuyama T (2009) NovQ is a prenyltransferase capable of catalyzing the addition of a dimethylallyl group to both phenylpropanoids and flavonoids. J Antibiot (Tokyo) 62: 385-392.
6. Seeger K, Flinspach K, Haug-Schifferdecker E, Kulik A, Gust B, et al. (2011) The biosynthetic genes for prenylated phenazines are located at two different chromosomal loci of *Streptomyces cinnamonensis* DSM 1042. Microb Biotechnol 4: 252-262.

7. Saleh O, Gust B, Boll B, Fiedler HP, Heide L (2009) Aromatic prenylation in phenazine biosynthesis: dihydrophenazine-1-carboxylate dimethylallyltransferase from *Streptomyces anulatus*. J Biol Chem 284: 14439-14447.
8. Haug-Schifferdecker E, Arican D, Brückner R, Heide L (2010) A new group of aromatic prenyltransferases in fungi, catalyzing a 2,7-dihydroxynaphthalene 3-dimethylallyl-transferase reaction. J Biol Chem 285: 16487-16494.
9. Kumano T, Richard SB, Noel JP, Nishiyama M, Kuzuyama T (2008) Chemoenzymatic syntheses of prenylated aromatic small molecules using *Streptomyces* prenyltransferases with relaxed substrate specificities. Bioorg Med Chem 16: 8117-8126.
10. Steffan N, Grundmann A, Yin WB, Kremer A, Li SM (2009) Indole prenyltransferases from fungi: a new enzyme group with high potential for the production of prenylated indole derivatives. Curr Med Chem 16: 218-231.
11. Markert A, Steffan N, Ploss K, Hellwig S, Steiner U, et al. (2008) Biosynthesis and accumulation of ergoline alkaloids in a mutualistic association between *Ipomoea asarifolia* (Convolvulaceae) and a Clavicipitalean Fungus. Plant Physiol 147: 296-305.
12. Wang J, Machado C, Panaccione DG, Tsai HF, Schardl CL (2004) The determinant step in ergot alkaloid biosynthesis by an endophyte of perennial ryegrass. Fungal Genet Biol 41: 189-198.
13. Unsöld IA, Li SM (2006) Reverse prenyltransferase in the biosynthesis of fumigaclavine C in *Aspergillus fumigatus*: gene expression, purification, and characterization of fumigaclavine C synthase FGAPT1. Chembiochem 7: 158-164.
14. Metzger U, Schall C, Zocher G, Unsöld I, Stec E, et al. (2009) The structure of dimethylallyl tryptophan synthase reveals a common architecture of aromatic prenyltransferases in fungi and bacteria. Proc Natl Acad Sci U S A 106: 14309-14314.
15. Jost M, Zocher G, Tarcz S, Matuschek M, Xie X, et al. (2010) Structure-function analysis of an enzymatic prenyl transfer reaction identifies a reaction chamber with modifiable specificity. J Am Chem Soc 132: 17849-17858.
16. Grundmann A, Kuznetsova T, Afiyatullov SS, Li S-M (2008) FtmPT2, an *N*-prenyltransferase from *Aspergillus fumigatus*, catalyses the last step in the biosynthesis of Fumitremorgin B. Chembiochem 9: 2059-2063.
17. Yin WB, Ruan HL, Westrich L, Grundmann A, Li S-M (2007) CdpNPT, an *N*-Prenyltransferase from *Aspergillus fumigatus*: Overproduction, Purification and Biochemical Characterisation. Chembiochem 8: 1154-1161.
18. Yin WB, Grundmann A, Cheng J, Li SM (2009) Acetylaszonalenin biosynthesis in *Neosartorya fischeri*. Identification of the biosynthetic gene cluster by genomic mining and functional proof of the genes by biochemical investigation. J Biol Chem 284: 100-109.
19. Kremer A, Westrich L, Li SM (2007) A 7-dimethylallyltryptophan synthase from *Aspergillus fumigatus*: overproduction, purification and biochemical characterization. Microbiology 153: 3409-3416.
20. Schneider P, Weber M, Hoffmeister D (2008) The *Aspergillus nidulans* enzyme TdiB catalyzes prenyltransfer to the precursor of bioactive asterriquinones. Fungal Genet Biol 45: 302-309.
21. Ding Y, Williams RM, Sherman DH (2008) Molecular analysis of a 4-dimethylallyltryptophan synthase from *Malbranchea aurantiaca*. J Biol Chem 283: 16068-16076.
22. Ding Y, de Wet JR, Cavalcoli J, Li S, Greshock TJ, et al. (2010) Genome-based characterization of two prenylation steps in the assembly of the stephacidin and notoamide anticancer agents in a marine-derived *Aspergillus* sp. J Am Chem Soc 132:

12733-12740.
23. Liu X, Walsh CT (2009) Characterization of cyclo-acetoacetyl-L-tryptophan dimethylallyltransferase in cyclopiazonic acid biosynthesis: substrate promiscuity and site directed mutagenesis studies. Biochemistry 48: 11032-11044.
24. Sanchez JF, Entwistle R, Hung JH, Yaegashi J, Jain S, et al. (2011) Genome-based deletion analysis reveals the prenyl xanthone biosynthesis pathway in *Aspergillus nidulans*. J Am Chem Soc.
25. Zou HX, Xie X, Zheng XD, Li SM (2010) The tyrosine *O*-prenyltransferase SirD catalyzes *O*-, *N*-, and *C*-prenylations. Appl Microbiol Biotechnol 89: 1443-1451.
26. Chooi YH, Cacho R, Tang Y (2010) Identification of the viridicatumtoxin and griseofulvin gene clusters from *Penicillium aethiopicum*. Chem Biol 17: 483-494.
27. Takahashi S, Takagi H, Toyoda A, Uramoto M, Nogawa T, et al. (2010) Biochemical characterization of a novel indole prenyltransferase from *Streptomyces* sp. SN-593. J Bacteriol 192: 2839-2851.
28. Schultz AW, Oh DC, Carney JR, Williamson RT, Udwary DW, et al. (2008) Biosynthesis and structures of cyclomarins and cyclomarazines, prenylated cyclic peptides of marine actinobacterial origin. J Am Chem Soc 130: 4507-4516.
29. Edwards DJ, Gerwick WH (2004) Lyngbyatoxin biosynthesis: sequence of biosynthetic gene cluster and identification of a novel aromatic prenyltransferase. J Am Chem Soc 126: 11432-11433.
30. Melzer M, Heide L (1994) Characterization of polyprenyldiphosphate: 4-hydroxybenzoate polyprenyltransferase from *Escherichia coli*. Biochim Biophys Acta 1212: 93-102.
31. Suvarna K, Stevenson D, Meganathan R, Hudspeth ME (1998) Menaquinone (vitamin K2) biosynthesis: localization and characterization of the *menA* gene from *Escherichia coli*. J Bacteriol 180: 2782-2787.
32. Savidge B, Weiss JD, Wong YH, Lassner MW, Mitsky TA, et al. (2002) Isolation and characterization of homogentisate phytyltransferase genes from *Synechocystis* sp. PCC 6803 and *Arabidopsis*. Plant Physiol 129: 321-332.
